# Narrative reviews

**DOI:** 10.4178/epih/e2014018

**Published:** 2014-09-11

**Authors:** Jong-Myon Bae

**Affiliations:** Department of Preventive Medicine, Jeju National University School of Medicine, Jeju, Korea

**Keywords:** Review literature as topic, Qualitative research, Qualitative evaluation, Meta-analysis as topic, Evidence-based practice

## Abstract

Although qualitative researches (QR) are invaluable in understanding complex healthcare situations, the quantitative systematic reviews could not treat them. To improve quality of healthcare services, results of QR should be considered in healthcare decision-making processes. Several methods and theories for synthesizing evidences of QR have been developed. In order to activate the narrative reviews and mixed methods reviews in Korean healthcare academies, I arranged the related nomenclatures and suggested some issues to conduct them.

## INTRODUCTION

In providing healthcare services, the evidence-based medicine (EBM) paradigm [1-3]—the idea that healthcare-related decisions should be made on the basis of the best evidence—is expanding to healthcare-related policy-making in nursing and health science as well as medicine [4-6]. Amidst this trend, quantitative systematic reviews is a new research approach. It is a term used to designate the approach to examining the effects of a treatment or an intervention adopting a statistical method called meta-analysis [7]. However, the results of qualitative research which cannot obtain quantitative outcomes, such as relative risk, odds ratio, and number needed to treat (NNT), tend to be ignored or excluded in the application of the systematic reviews process [8].

Not only the definition of qualitative research, but also the characteristic comparisons between qualitative and quantitative research are well documented in the papers by Murphy et al. [9] and Draper [10]. In short, qualitative research can be a useful guide for understanding complicated situations in the real world and can serve as groundwork for new hypotheses [11-15]. Consequently, efforts to use the synthesis of qualitative research results as the grounds for decision-making were already underway in 1990s when EBM emerged [16-23]. However, recently, in line with the trend toward improving medical treatment quality through patient-centered and evidence-based diagnostic and therapeutic services [24,25], efforts for complementary utilization of qualitative research results are experiencing a revival [13, 15,26-34]. In particular, the claim that both quantitative and qualitative research should be utilized for proper understanding of overall healthcare problems is gaining influence. [18,35,36].

Meanwhile, because the synthesis of qualitative research results is inevitably different from a quantitative systematic reviews, there have been many attempts to overcome this gap [8, 37-40]. For this reason, I would like to systemize the attempts to synthesize qualitative research results undertaken so far. This work is expected to show the framework of the discussions related to qualitative research and lay a cornerstone for the vitalization of systematic reviews on qualitative research in South Korea.

## MAIN BODY

### Terms related to the synthesis of qualitative research

Among the theories about qualitative research presented so far, works of Draper [10], Barnett-Page & Thomas [41], Dixon-Woods et al. [42], and Thorne et al. [43] may be representative. I arranged various terms related to qualitative research, grouping them according to the emphasis intended.Appendix 1 is the overview of the healthcare research applying this nomenclature. At a glance, we can see many terms are suggested with the emphasis placed on the meaning of the term “synthesis” of qualitative research results in contrast to the application of the meta-analytic statistical method of quantitative research results. In particular, we can see the term “mixed methods research” has often been applied recently in the attempt to include both quantitative and qualitative research [44].

### Process of synthesis of qualitative research

As can be seen in Appendix 1, the high number of related terms implies that the establishment of the relevant research methodology is a difficult task [45]. Sinuff et al. [46] shows the difference between quantitative and qualitative research processes in a diagram. However, a look at the suggested processes in relation to qualitative research synthesis [34,45,47-54] reveals that they stick to the big frame of “Ask - Acquire - Appraise - Apply - Assess,” 5A of the evidence cycle, although they show a certain diversity [55].

Searches of qualitative research to collect a body of related literature are more difficult than quantitative research [56-58]. This is because of different database services and the need to search for gray literature that has been issued but not officially published and made available in market, such as reports published by institutions or academic narrative reports [8]. It is also attributable to the need to resort, in addition to securing lists through search formulae, to hand searching which involves bibliography browsing in search of related papers and snowballing searching which traces one paper after another in chronological order [14,59].

Because of the diversity of research methods and fields of application, qualitative research does not easily lend itself to standardizing the items of qualitative evaluation in the literature of interest [8,59-64]. Nevertheless, the following achievements harvested so far deserve to be listed: (1) Thomas et al. [65] suggested evaluation items which matched quantitative-qualitative research. (2) Clark [66] developed ‘RATS’ evaluation tool, which is an acronym for Relevance, Appropriateness, Transparency, and Soundness. (3) Daly et al. [67] suggested a stratified structure by the contents of qualitative research. (4) Rodgers et al. [34] applied the EPPI approach that evaluates the persuasive power of evidence. And (5) Dixon-Woods et al. [68] suggested a tool called CASP (Critical Appraisal Skills Programme tool) which is composed of 10 items.

For the synthesis of evaluation results, a best fit frameworks is established [65,69-73] or a simulations model is selected [74]. The commercial program called NVivo has been developed [52, 75,76]. Feasibility research examining the applicability of this program in Korean society needs to be accumulated.

Also, a reporting guideline named RAMESES (Realist And MEta-narrative Evidence Synthesis: Evolving Standards) has been developed for application when the research results of systematic reviews of qualitative research are to be reported in papers [77,78]. Making a flow chart is also suggested in cases where the mixed methods reviews approach is adopted which involves both quantitative and qualitative research [79,80].

## CONCLUSION AND PROPOSAL

There are few systematic reviews on qualitative research in healthcare-related scholarship in South Korea. For the qualitative improvement of healthcare in this scarce situation, research in various healthcare fields should be conducted. Therefore, I suggest three things as below.

First, establishing the nomenclature of systematic reviews on qualitative research is an urgent task, because systematic and coherent use of well-established terms is important when a multitude of suggested terms are in use, as shown in Appendix 1. The synthesis of quantitative research through the meta-analytic statistical method can be termed ‘quantitative systematic reviews’ and that of qualitative research, ‘qualitative systematic reviews’ [81]. However, given the current situation that systematic reviews have been established as the major research methodology of the synthesis of the evidences of quantitative research and the meta-analysis applied to this is recognized as statistical methodology [81], systematic reviews in the narrow sense mean quantitative systematic reviews [82]. On the contrary, qualitative systematic reviews is also called narrative systematic reviews and recently in a more abbreviated form called “narrative reviews” [29,83-85]. However, the term ‘meta-narrative reviews’ [77,78] does not fit and its use should be avoided because the meta-analysis corresponding to this is a statistical method, not a research method [81]. Therefore, I suggest a terminological differentiation between (quantitative) systematic reviews and (qualitative) narrative reviews, whereby the term “mixed methods reviews” may be used when both quantitative and qualitative research are involved. It was in consideration of this point that I titled this study “Narrative Reviews.”

Second, the practical way to revitalize narrative reviews research when Korean researchers do not have much experience conducting narrative reviews is the critical reading of good research cases from various academic fields and their application to practical use. To facilitate this process, I organized useful research cases by academic field and presented them in Appendix 2.

Third, there is a need to establish a research-supporting organization and expand research human resources for qualitative research in the process of planning and conducting clinical studies [15]. The basic prior condition of proper narrative reviews is good results from qualitative research. First and foremost, given the fact that multidisciplinary cooperation is of vital importance for qualitative research, an organizational reshuffling appears necessary to facilitate efficient cooperation.

## Appendix 1. Summary tables of nomenclatures about methodologies for synthesis of qualitative researches

**Table:**

To	Nomenclatures	[References]
Emphasize qualitative synthesis	Narrative summary	[A01, A02]
	Thematic synthesis	[A03]
	Textual narrative synthesis	[A04]
	Critical interpretive synthesis	[A05, A06]
	Framework synthesis	[A07]
	Realist synthesis	[A08, A09]
Contrast quantitative systematic review	Qualitative systematic review	[A10,A11]
	Synthesis of qualitative research	[A12]
	Narrative systematic review	[A13]
	Qualitative comparative analysis	[A14, A15]
	Qualitative meta-synthesis	[A16]
	Qualitative meta-summary	[A17]
Contrast meta-analysis	Meta-synthesis	[A18, A19]
	Meta-narrative review	[A20, A21]
	Meta-ethnography	[A22, A23]
	Meta-study	[A24]
	Meta-interpretation	[A25]
Include quantitative & qualitative researches	Mixed methods research	[A26-A29]
	Ecological triangulation	[A01]

## Appendix 2. Some articles related to qualitative researches about health care services

**Table:**

Domain	[References]
Preventive medicine	Pharmacoepidemiology [B01, B02]
	Occupation [B03]
	Hospital management [B04]
Clinical medicine	Obstetrics [B05]
	Hospice [B06]
	Patient-doctor relationship [B07]
Nurse	Diabetic care [B08, B09]
	Insight into cancer [B10]
Health promotion	Mental health [B11]
	School health [B12-B15]
	Health program [B16-B20]
	Vaccine program [B21]
Health policy	Policy decision [B22]
	Control plan of tuberculosis [B23]
