# Supplementary material for: Narrative reviews
Source: Epidemiol Health. 2014 Sep 11;36:e2014018. doi: 10.4178/epih/e2014018 (PMC4207097; doi:10.4178/epih/e2014018)
Supplement: Supplementary file 1 [file epih-36-e2014018-supplementary.pdf]

<연구 방법론>

서술적 고찰

Narrative reviews

Abstract

Although qualitative researches (QR) are invaluable in understanding complex healthcare situations, the quantitative systematic review could not treat them. To improve quality of healthcare services, results of QR should be considered in healthcare decision-making processes. Several methods and theories for synthesizing evidences of QR have been developed. In order to activate the narrative reviews and mixed methods reviews in Korean healthcare academies, I arranged the related nomenclatures and suggested some issues to conduct them.

Key words: Review literature as topic, Qualitative research, Qualitative evaluation, Meta-analysis as topic, Evidence-based Practice

<한글초록>

질적연구는 보건의료의 복잡성을 이해하는데 매우 유용함에도 불구하고, 메타분석을 수행하는 체계적 고찰에서는 다루어지지 않는다. 그렇지만 보건의료의 질적 향상을 도모하려면 질적 연구의 결과를 보건의료 결정과정에서 반영해야 한다. 질적 연구 결과들을 합성하려는 여러 시도들이 있어온 것을 요약하고, 관련한 용어들을 정리하여 질적연구와 이를 합성하는 서술적 고찰이 활성화되는 바탕을 마련코자 한다.

1. 서론

보건의료서비스 제공에 있어 최선의 근거에 따라 결정하자는 근거중심의학 (Evidence-based medicine, EBM)의 패러다임[1-3]은 의학뿐만 아니라 간호학, 보건학 나아가 보건정책의 의사결정에까지 확대되고 있다 [4-6]. 이런 추세 속에 치료나 개입의 효과를 메타분석이란 통계방법을 적용한 양적 체계적 고찰 (quantitative systematic review)이 새로운 연구방법으로 자리잡게 되었다 [7]. 그런데 상대위험도 (Relative risk), 비교위험도 (odds ratio), NNT (number needed to treatment)등의 계량적 결과를 얻어낼 수 없는 질적 연구 (qualitative research)의 결과들은 체계적 고찰 적용과정에서 무시되거나 제외되는 경향이 나타났다 [8].

질적 연구의 정의뿐만 아니라, 양적 연구와의 특성 비교는 Murphy et al. [9]와 Draper [10]의 논문에 잘 제시되어 있다. 요약하자면 질적 연구는 현실세계의 복잡한 상황을 이해할 수 있게 해주고, 새로운 가설을 제시해 주는 유용한 근거가 된다는 것이다 [11-15]. 이에 따라 질적 연구 결과를 합성 (synthesis)하여 의사결정의 근거로 삼으려는 노력은 EBM이 대두되는 1990년대에도 이미 있어 왔다 [16-23]. 그런데 최근 들어 환자중심 (patient-centered)과 근거바탕 (evidence-based)의 진료를 해서 의료의 질을 향상시키려는 움직임 [24,25]에 맞추어, 질적 연구 결과를 상보적으로 활용하려는 노력이 다시 제기되고 있다 [13, 15, 26-34]. 특히 양적-질적 연구 결과들을 모두 활용해야 보건의료 전반의 문제를

제대로 이해할 수 있다는 주장이 힘을 얻고 있다 [18,35,36].

그런데 질적 연구결과를 합성 (synthesis)하는 것은 당연히 양적 체계적 고찰과 다를 수밖에 없기 때문에, 이를 극복하고자 수많은 시도들이 있어 왔다 [8, 37-40]. 이에 저자는 질적 연구결과의 합성에 관한 그동안의 시도들을 정리해 보고자 한다. 이러한 작업은 질적 연구와 관련한 논의를 위한 기초 틀을 제시하는 것이며, 국내에서 질적 연구에 대한 체계적 고찰연구가 활성화 될 바탕을 마련할 것으로 기대한다.

2. 본론

가. 질적 연구 합성 관련 용어들

지금까지의 질적 연구에 관한 이론들은 Draper [10], Barnett-Page & Thomas [41], Dixon-Woods et al. [42], Thorne et al. [43] 등의 논문을 참고할 수 있다. 그리고 질적 연구와 관련한 다양한 용어들이 강조하려는 의미에 맞추어 정리한 다음, 이를 실제 적용한 보건의료 연구들을 부록 1로 제시하였다. 일견할 때, 양적 연구결과의 메타분석 통계법을 적용한 것과 대조하여 질적 연구 결과의 합성이란 의미를 부각하면서 여러 용어들이 제시된 것을 알아낼 수 있다. 특히 양적 연구와 질적 연구를 모두 포함하여 고찰을 하려는 시도에서 Mixed methods research 용어가 최근 들어 활발히 적용되고 있다는 점이다 [44].

나. 질적 연구 합성 수행 과정

부록 1에서 보듯 관련한 용어들이 많다는 것은 관련한 연구방법론을 정립한다는 것이 난제라는 것을 의미한다 [45]. 양적 연구와 질적 연구의 수행 과정의 차이점을 Sinuff et al. [46]은 도식화해서 보여주고 있다. 그런데 질적 연구 합성에 관련하여 수행과정을 제안한 것들 [34, 45, 47-54]을 살펴보면, 비록 다양하지만 근거 순환 (evidence cycle)의 5A 인 Ask – Acquire – Appraise – Apply – Assess 라는 큰 골격에서 벗어나지 않고 있다 [55].

관련 문헌 확보를 위한 질적 연구의 검색은 양적 연구보다 더 까다롭다 [56-58]. 제공하는 검색 데이터베이스도 달라질 뿐만 아니라, 기관발간 보고서나 학술적 기술보고서 같이 발행은 되었지만 정식으로 출판되어 시판되는 것이 아닌 회색 문헌(gray literature)도 찾아야 할 필요가 있기 때문이다 [8]. 그리고 검색식을 이용한 목록확보뿐만 아니라, 관련 논문의 참고문헌 목록을 다시 뒤져야 하는 hand searching과 관련 논문에서 논문으로 꼬리를 물고 추적하여 찾아내는 snowballing searching 기법을 추가로 동원해야 하기 때문이다 [14,59].

질적 연구는 적용하는 연구 분야뿐만 아니라 연구 방법도 다양하여 해당 문헌의 질적 평가 항목을 표준화하기도 쉽지 않다 [8, 59-64]. 그렇지만 지금까지 의미 있는 성과들을 나열하자면 다음과 같다. (1) Thomas et al. [65]은 양적-질적 연구에 맞추어 평가 항목을 제시하였으며 (2) Clark [66]은 Relevance, Appropriateness, Transparency, Soundness 의 앞 자를 딴 RATS 평가도구를 개발하였고, (3) Daly et al. [67]는 질적 연구 내용에 따른 계층 구조를 제시하였으며 (4) Rodgers et al. [34]는 질적 연구들의 합성을 수행하는 가이드라인을 개발하였고 (5) Dixon-Woods et al. [68]은 10개의 항목으로 된 CASP (Critical Appraisal Skills Programme tool)란 도구를 제시하였다.

평가결과들의 합성을 위해서 최선의 해석을 해낼 수 있는 설명 틀 (best fit framework)이 구축되거나 [65, 69-73], 시뮬레이션 모델 (simulation model)이 선정되고 있다

[74]. 그리고 NVivo 란 상용 프로그램이 개발되어 있는데 [52,75,76], 한국 사회에 얼마나 맞을 지에 대한 타당성 연구가 축적될 필요가 있다.

질적 연구의 체계적 고찰을 통한 연구 결과를 논문화할 때 적용하도록 RAMESES (Realist And MEta-narrative Evidence Synthesis: Evolving Standards) 이름의 보고지침이 개발되어 있다 [77,78]. 만약 양적 연구와 질적 연구를 모두 포함한 Mixed method review인 경우 흐름도 작성도 제시되어 있다 [79,80].

3. 결어 및 제언

한국의 보건의료학계에서 질적 연구의 체계적 고찰 수행 연구를 찾기가 어려운 것이 현실이다. 이런 척박한 상황에서 보건의료의 질적 향상을 위한 다양한 보건의료 영역에서의 연구가 수행될 필요가 있다. 이를 위하여 아래의 세 가지 제언을 한다.

첫째, 질적 연구의 체계적 고찰에 관한 용어 정립이 시급하다. 부록 1에서 보듯이 많은 용어들이 제시되어 있는 상황에서 분명한 뜻을 가진 용어를 일관되게 사용하는 것 중요하기 때문이다. 양적 연구들을 메타분석 통계법으로 합성할 경우를 quantitative systematic reviews 라 하고, 이와 대비하여 질적 연구들의 합성을 qualitative systematic reviews라 할 수 있다 [81]. 그런데 현 시점에서 체계적 고찰 (systematic reviews)은 양적 연구의 근거 합성의 주된 연구 방법론으로 정착되어 있으며, 이때 적용하는 메타분석 (meta-analysis)는 통계 방법론으로 더 많이 인식하고 있다는 점 [81]을 감안한다면 좁은 의미의 체계적 고찰은 quantitative systematic reviews를 의미하고 있다 [82]. 반면에 qualitative systematic reviews는 narrative systematic reviews라고도 하는데, 이를 줄여서 최근에는 narrative reviews (서술적 고찰)라 부르고 있다 [29, 83-85]. 그런데 meta-narrative reviews라고 언급하는 것[77,78]은 삼갔으면 하는 것은, 이와 대응하는 meta-analysis 는 통계분석방법이지 연구방법이 아니기 때문이다 [81]. 따라서 (양적) 체계적 고찰과 (질적) 서술적 고찰의 두 용어로 구분하면서, 만약 양적-질적 연구 모두를 다룰 경우는 Mixed methods reviews (복합적 고찰)로 연구방법상의 명칭들을

정리하기를 제안한다. 이를 염두에 두고 본 논문의 제목을 '서술적 고찰'이라 제시하였다.

둘째, 국내 연구진의 서술적 고찰에 대한 수행 경험이 일천한 상황에서, 서술적 고찰연구의 활성화를 도모하는 현실적 방안은, 다방면의 학문적 영역에 걸쳐 이루어진 좋은 연구 사례들을 숙독하고 이를 실제 적용하는 경험을 하는 것이다. 도움이 되는 연구 사례를 학문 영역별로 정리하여 부록 2로 제시하였다.

셋째, 임상연구를 계획하고 수행하는 과정에서 질적 연구를 수행할 수 있는 연구 지원 조직 구축과 연구 인력 양성이 필요하다 [15]. 서술적 고찰이 제대로 이루어지기 위한 근본적인 전제조건은 좋은 질적 연구 결과가 있어야 하기 때문이다. 특히 질적 연구는 다학제간 협조가 필수적이란 점에서 효과적인 협력이 이루어질 수 있는 조직으로 개편하는 것이 꼭 필요하겠다.

감사의 글

이 논문은 2014학년도 제주대학교 학술진흥연구비 지원사업에 의하여 연구되었음

참고문헌

1. Miles A, Bentley P, Polychronis A, Grey J. Evidence-based medicine: why all the fuss? This is why. J Eval Clin Pract 1997;3:83-86.
2. Miles A, Bentley P, Polychronis A, Grey J, Price N. Recent progress in health services research: on the need for evidence-based debate. J Eval Clin Pract 1998;4:257-265.
3. Miles A, Bentley P, Polychronis A, Grey J, Price N. Advancing the evidence-based healthcare debate. J Eval Clin Pract. 1999 May;5(2):97-101.
4. King S, Forbes K, Hanks GW, Ferro CJ, Chambers EJ. A systematic review of the use of opioid medication for those with moderate to severe cancer pain and renal impairment: A European Palliative Care Research Collaborative opioid guideline project. Palliat Med 2011;25:525.
5. Fox DM. Evidence of evidence-based health policy: the politics of systematic reviews in coverage decisions. Health Aff 2005;24:114-122.
6. Greenhalgh T, Wong G, Westhorp G, Pawson R. Protocol--realist and meta-narrative evidence synthesis: evolving standards (RAMESES). BMC Med Res Methodol 2011;11:115.
7. Bae JM. Methodological issues for determining intervals of subsequent cancer screening. Epidemiol Health 2014;36;e201410.
8. Jones K. Mission drift in qualitative research, or moving toward a systematic review of qualitative studies, moving back to a more systematic narrative review. Qualitative Report 2004;9:95-112.
9. Murphy E, Dingwall R, Greatbatch D, Parker S, Watson P. Qualitative research methods in health technology assessment: a review of the literature. Health Technol Assess 1998;2:1-274.
10. Draper AK. The principles and application of qualitative research. Proc Nutr Soc 2004;63:641-646.
11. Jones R. Why do qualitative research? BMJ 1995;311:2.
12. Murthy GV, Gupta SK. Qualitative research in ophthalmic sciences. Indian J Ophthalmol 1999;47:257-264.
13. Sofaer S. Qualitative methods: what are they and why use them? Health Serv Res 1999;34(5 Pt

2):1101-1118.

14. Greenhalgh T, Heath I. Measuring quality in the therapeutic relationship –Part 1: objective approaches. Qual Saf Health Care 2010;19:475-478.

15. Rapport F, Storey M, Porter A, Snooks H, Jones K, Peconi J, et al. Qualitative research within trials: developing a standard operating procedure for a clinical trials unit. Trials 2013;14:54.

16. Popay J, Rogers A, Williams G. Rationale and standards for the systematic review of qualitative literature in health services research. Qual Health Res 1998;8:341-351.

17. Barbour RS. Mixing qualitative methods: quality assurance or qualitative quagmire? Qual Health Res 1998;8:352-361.

18. McDowell I, MacLean L. Blending qualitative and quantitative study methods in health services research. Health Informatics J 1998;4:15-22.

19. Green J, Britten N. Qualitative research and evidence based medicine. BMJ 1998;316:1230-1232.

20. Harding G, Gantley M. Qualitative methods: beyond the cookbook. Fam Pract 1998;15:76-79.

21. Popay J, Williams G. Qualitative research and evidence-based healthcare. J R Soc Med 1998;91 Suppl 35:32-37.

22. Dixon-Woods M, Fitzpatrick R. Qualitative research in systematic reviews. Has established a place for itself. BMJ 2001;323:765-766.

23. Donovan J, Mills N, Smith M, Brindle L, Jacoby A, Peters T, et al. Quality improvement report: Improving design and conduct of randomised trials by embedding them in qualitative research: ProtecT (prostate testing for cancer and treatment) study. Commentary: presenting unbiased information to patients can be difficult. BMJ 2002;325:766-770.

24. Bae JM. Global trends in the use of nationwide big data for solving healthcare problems. J Korean Med Assoc 2014;57:386-390. (Korean)

25. Mohammed K, Nolan MB, Rajjo T, Shah ND, Prokop LJ, Varkey P, et al. Creating a Patient-Centered Health Care Delivery System: A Systematic Review of Health Care Quality From the Patient

Perspective. Am J Med Qual 2014 [PMID:25082873].

26. Pawson R, Greenhalgh T, Harvey G, Walshe K. Realist review--a new method of systematic review designed for complex policy interventions. J Health Serv Res Policy 2005;10 Suppl 1:21-34.

27. Barbour RS. The case for combining qualitative and quantitative approaches in health services research. J Health Serv Res Policy 1999;4:39-43.

28. Morgan DL. Practical strategies for combining qualitative and quantitative methods: applications to health research. Qual Health Res 1998;8:362-376.

29. Collins JA, Fauser BC. Balancing the strengths of systematic and narrative reviews. Hum Reprod Update 2005;11:103-104.

30. Moffatt S, White M, Mackintosh J, Howel D. Using quantitative and qualitative data in health services research - what happens when mixed method findings conflict? [ISRCTN61522618]. BMC Health Serv Res 2006;6:28.

31. Martin M. The need for an overall strategy. J Health Serv Res Policy 2005;10 Suppl 1:49-50.

32. Flemming K. The synthesis of qualitative research and evidence-based nursing. Evid Based Nurs 2007;10:68-71.

33. Jack SM. Utility of qualitative research findings in evidence-based public health practice. Public Health Nurs 2006;23:277-283.

34. Rodgers M, Sowden A, Petticrew M, Arai L, Roberts H, Britten N, et al. Testing methodological guidance on the conduct of narrative synthesis in systematic reviews: effectiveness of interventions to promote smoke alarm owenership and function. Evaluation 2009;15:49.

35. Black N. Why we need qualitative research. J Epidemiol Community Health 1994;48:425-426.

36. Berwick DM. The science of improvement. JAMA 2008;299:1182-1184.

37. Hoddinott P, Pill R. A review of recently published qualitative research in general practice. More methodological questions than answers? Fam Pract 1997;14:313-319.

38. Barbour RS. Checklists for improving rigour in qualitative research: a case of the tail wagging the

dog? BMJ 2001;322:1115-1117.
39. Dixon-Woods M, Fitzpatrick R, Roberts K. Including qualitative research in systematic reviews: opportunities and problems. J Eval Clin Pract 2001;7:125-133.
40. Lewin S, Glenton C, Oxman AD. Use of qualitative methods alongside randomised controlled trials of complex healthcare interventions: methodological study. BMJ 2009;339:b3496.
41. Barnett-Page E, Thomas J. Methods for the synthesis of qualitative research: a critical review. BMC Med Res Methodol 2009;9:59.
42. Dixon-Woods M, Agarwal S, Jones D, Young B, Sutton A. Synthesising qualitative and quantitative evidence: a review of possible methods. J Health Serv Res Policy 2005;10:45-53.
43. Thorne S, Jensen L, Kearney MH, Noblit G, Sandelowski M. Qualitative metasynthesis: reflections on methodological orientation and ideological agenda. Qual Health Res 2004;14:1342-65.
44. O'Cathain A, Murphy E, Nicholl J. Why, and how, mixed methods research is undertaken in health services research in England: a mixed methods study. BMC Health Serv Res 2007;7:85.
45. Forbes A, Griffiths P. Methodological strategies for the identification and synthesis of 'evidence' to support decision-making in relation to complex healthcare systems and practices. Nurs Inq 2002;9:141-155.
46. Sinuff T, Cook DJ, Giacomini M. How qualitative research can contribute to research in the intensive care unit. J Crit Care 2007;22:104-111.
47. Greenhalgh T, Robert G, Macfarlane F, Bate P, Kyriakidou O. Diffusion of innovations in service organizations: systematic review and recommendations. Milbank Q 2004;82:581-629.
48. Abu Dabrh AM, Firwana B, Cowl CT, Steinkraus LW, Prokop LJ, Murad MH. Health assessment of commercial drivers: a meta-narrative systematic review. BMJ Open 2014;4:e003434.
49. Mays N, Pope C, Popay J. Systematically reviewing qualitative and quantitative evidence to inform management and policy-making in the health field. J Health Serv Res Policy 2005;10 Suppl 1:6-20.

50. Flemming K. Synthesis of quantitative and qualitative research: an example using Critical Interpretive Synthesis. J Adv Nurs 2010;66:201-217.

51. Walsh D, Downe S. Meta-synthesis method for qualitative research: a literature review. J Adv Nurs 2005;50:204-211.

52. Leamy M, Bird V, Le Boutillier C, Williams J, Slade M. Conceptual framework for personal recovery in mental health: systematic review and narrative synthesis. Br J Psychiatry 2011;199:445-452.

53. Harden A, Garcia J, Oliver S, Rees R, Shepherd J, Brunton G, et al. Applying systematic review methods to studies of people's views: an example from public health research. J Epidemiol Community Health 2004;58:794-800.

54. Tricco AC, Tetzlaff J, Moher D. The art and science of knowledge synthesis. J Clin Epidemiol 2011;64:11-20.

55. Bae JM, Park BJ, Ahn YO. Perspectives of clinical epidemiology in Korea. J Korean Med Assoc 2013;56:718-723. (korean)

56. Evans D. Database searches for qualitative research. J Med Libr Assoc 2002;90:290-293.

57. Flemming K, Briggs M. Electronic searching to locate qualitative research: evaluation of three strategies. J Adv Nurs 2007;57:95-100.

58. Shaw RL, Booth A, Sutton AJ, Miller T, Smith JA, Young B, et al. Finding qualitative research: an evaluation of search strategies. BMC Med Res Methodol 2004;4:5.

59. Methley AM, Chew-Graham C, Campbell S, Cheraghi-Sohi S. Experiences of UK health-care services for people with Multiple Sclerosis: a systematic narrative review. Health Expect 2014 [PMID: 24990077].

60. Mays N, Pope C. Qualitative research in health care. Assessing quality in qualitative research. BMJ 2000;320:50-52.

61. Dixon-Woods M, Shaw RL, Agarwal S, Smith JA. The problem of appraising qualitative research. Qual Saf Health Care 2004;13:223-225.

62. Reynolds J, Kizito J, Ezumah N, Mangesho P, Allen E, Chandler C. Quality assurance of qualitative research: a review of the discourse. Health Res Policy Syst 2011;9:43.

63. Boeije HR, van Wesel F, Alisic E. Making a difference: towards a method for weighing the evidence in a qualitative synthesis. J Eval Clin Pract 2011;17:657-663.

64. Crossland L, Janamian T, Jackson CL. Key elements of high-quality practice organisation in primary health care: a systematic review. Med J Aust 2014;201:S47-S51.

65. Thomas J, Harden A, Oakley A, Oliver S, Sutcliffe K, Rees R, et al. Integrating qualitative research with trials in systematic reviews. BMJ 2004;328:1010-1012.

66. Clark JP. How to peer review a qualitative manuscript. In: Godlee F, Jefferson T, editors. Peer review in health sciences. 2nd ed. Londo: BMJ Books; 2003, p.219-235.

67. Daly J, Willis K, Small R, Green J, Welch N, Kealy M, et al. A hierarchy of evidence for assessing qualitative health research. J Clin Epidemiol 2007;60:43-49.

68. Dixon-Woods M, Sutton A, Shaw R, Miller T, Smith J, Young B, et al. Appraising qualitative research for inclusion in systematic reviews: a quantitative and qualitative comparison of three methods. J Health Serv Res Policy 2007;12:42-47.

69. Carroll C, Booth A, Leaviss J, Rick J. "Best fit" framework synthesis: refining the method. BMC Med Res Methodol 2013;13:37.

70. Lemire M, Demers-Payette O, Jefferson-Falardeau J. Dissemination of performance information and continuous improvement: A narrative systematic review. J Health Organ Manag 2013;27:449-478.

71. Jacobson N, Butterill D, Goering P. Development of a framework for knowledge translation: understanding user context. J Health Serv Res Policy 2003;8:94-99.

72. Oliver SR, Rees RW, Clarke-Jones L, Milne R, Oakley AR, Gabbay J, et al. A multidimensional conceptual framework for analysing public involvement in health services research. Health Expect 2008;11:72-84.

73. Eakin JM, Mykhalovskiy E. Reframing the evaluation of qualitative health research: reflections on a review of appraisal guidelines in the health sciences. J Eval Clin Pract 2003;9;187-194.

74. Fone D, Hollinghurst S, Temple M, Round A, Lester N, Weightman A, et al. Systematic review of the use and value of computer simulation modelling in population health and health care delivery. J Public Health Med 2003;25;325-335.

75. QSR International. NVivo 10 for Windows [cited 2014 Sep 3]. Available from: http://www.qsrinternational.com/products_nvivo.aspx

76. Noble H, Smith J. Qualitative data analysis: a practical example. Evid Based Nurs 2014;17:2-3.

77. Wong G, Greenhalgh T, Westhorp G, Buckingham J, Pawson R. RAMESES publication standards: meta-narrative reviews. BMC Med 2013;11:20.

78. Wong G, Greenhalgh T, Westhorp G, Buckingham J, Pawson R. RAMESES publication standards: meta-narrative reviews. J Adv Nurs 2013;69:987-1004.

79. Gough D, Thomas J, Oliver S. Clarifying differences between review designs and methods. Syst Rev 2012;1:28.

80. Sandelowski M, Voils CI, Leeman J, Crandell JL. Mapping the Mixed Methods-Mixed Research Synthesis Terrain. J Mix Methods Res 2012;6:317-331.

81. Crombie IK, Davies HTO. What is meta-analysis? [cited 2014 Sep 3]. Available from: http://www.whatisseries.co.uk/what-is-meta-analysis/.

82. Ahn HS, Kim HJ. An introduction to systematic review. J Korean Med Assoc 2014;57:49-59.

83. Del Pozzo-Magaña BR, Rieder MJ, Lazo-Langner A. Quality of life in children with adverse drug reactions: a narrative and systematic review. Br J Clin Pharmacol 2014 [PMID:24833305]

84. Cipriani A, Geddes J. Comparison of systematic and narrative reviews: the example of the atypical antipsychotics. Epidemiologia e psichiatria sociale 2003;12:146-153.

85. Icahn School of Medicine at Mount Sinai. EBM skills on the wards [cited 2014 Sep 3]. Available from: http://libguides.mssm.edu/content.php?pid=417116&sid=3882519.

Appendix 1. Summary tables of nomenclatures about methodologies for synthesis of qualitative research

To	Nomenclatures	related references*
Emphasize Qualitative Synthesis	Narrative summary	[A01 (1990)] [A02 (2000)]
	Thematic synthesis	[A03 (2008)]
	Textual narrative synthesis	[A04 (2007)]
	Critical interpretive synthesis	[A05 (2006)] [A06 (2010)]
	Framework synthesis	[A07 (2008)]
	Realist synthesis	[A08 (2002)] [A09 (2005)]
Contrast Quantitative Systematic Review	Qualitative systematic review	[A10 (2007)] [A11 (2009)]
	Synthesis of qualitative research	[A12 (2009)]
	Narrative systematic review	[A13 (2009)]
	Qualitative comparative analysis	[A14 (1992)] [A15 (2000)]
	Qualitative meta-synthesis	[A16 (2004)]
	Qualitative meta-summary	[A17 (2009)]
Contrast Meta-analysis	Meta-synthesis	[A18 (1997)] [A19 (2005)]
	Meta-narrative review	[A20 (2004)] [A21 (2014)]
	Meta-ethnography	[A22 (2003)] [A23 (2011)]
	Meta-study	[A24 (2002)]
	Meta-interpretation	[A25 (1999)]

Include Quantitative & Qualitative researches	Mixed methods research	[A26 (2006)] [A27 (2007)] [A28 (2012)] [A29 (2012)]
	Ecological triangulation	[A01 (2008)]

* Reference number (publication year)

A01. Abbott A. Conceptions of time and events in social science methods: causal and narrative approaches. Historical Methods 100;23:140-150.

A02. Fairbank L, O'Meara S, Renfrew MJ, Woolridge M, Sowden AJ, Lister-Sharp D. A systematic review to evaluate the effectiveness of interventions to promote the initiation of breastfeeding. Health Technol Assess 2000;4:1-171.

A03. Thomas J, Harden A. Methods for the thematic synthesis of qualitative research in systematic reviews. BMC Med Res Methodol 2008;8:45.

A04. Lucas PJ, Baird J, Arai L, Law C, Roberts HM. Worked examples of alternative methods for the synthesis of qualitative and quantitative research in systematic reviews. BMC Med Res Methodol 2007;7:4.

A05. Dixon-Woods M, Cavers D, Agarwal S, Annandale E, Arthur A, Harvey J, et al. Conducting a critical interpretive synthesis of the literature on access to healthcare by vulnerable groups. BMC Med Res Methodol 2006;6:35.

A06. Flemming K. Synthesis of quantitative and qualitative research: an example using Critical Interpretive Synthesis. J Adv Nurs 2010;66:201-217.

A07. Oliver SR, Rees RW, Clarke-Jones L, Milne R, Oakley AR, Gabbay J, et al. A multidimensional conceptual framework for analysing public involvement in health services research. Health Expect 2008;11:72-84.

A08. Pawson R. Evidence-based policy: the promise of 'Realist Synthesis'. Evaluation 2002;8:340-358.

A09. Pawson R, Greenhalgh T, Harvey G, Walshe K. Realist review--a new method of systematic review designed for complex policy interventions. J Health Serv Res Policy 2005;10 Suppl 1:21-34.

A10. Flemming K. Synthesis of qualitative research and evidence-based nursing. Br J Nurs 2007;16:616-620.

A11. Timulak L. Meta-analysis of qualitative studies: a tool for reviewing qualitative research findings in psychotherapy. Psychother Res 2009;19:591-600.

A12. Barnett-Page E, Thomas J. Methods for the synthesis of qualitative research: a critical review. BMC Med Res Methodol 2009;9:59.

A13. Roundtree AK, Kallen MA, Lopez-Olivo MA, Kimmel B, Skidmore B, Ortiz Z, et al. Poor reporting of search strategy and conflict of interest in over 250 narrative and systematic reviews of two biologic agents in arthritis: a systematic review. J Clin Epidemiol 2009;62:128-137.

A14. Haggerty TR. Unravelling patterns of multiple conjunctural causation in comparative research: Ragin's qualitative comparative method. J Comparative Physical Edu Sport 1992;14:19-27.

A15. Cress DM, Snow DA. The outcomes of homeless mobilization: The influence of organization, disruption, political mediation, and framing. Am J Soc 2000;105:1063-1104.

A16. Thorne S, Jensen L, Kearney MH, Noblit G, Sandelowski M. Qualitative metasynthesis: reflections on methodological orientation and ideological agenda. Qual Health Res 2004;14:1342-65.

A17. Barnett-Page E, Thomas J. Methods for the synthesis of qualitative research: a critical review. BMC Med Res Methodol 2009;9:59.

A18. Sandelowski M, Docherty S, Emden C. Focus on qualitative methods. Qualitative metasynthesis: issues and techniques. Res Nurs Health 1997;20:365-371.

A19. Walsh D, Downe S. Meta-synthesis method for qualitative research: a literature review. J Adv Nurs 2005;50:204-211.

A20. Greenhalgh T, Robert G, Macfarlane F, Bate P, Kyriakidou O. Diffusion of innovations in service

organizations: systematic review and recommendations. Milbank Q 2004;82:581-629.

A21. Abu Dabrh AM, Firwana B, Cowl CT, Steinkraus LW, Prokop LJ, Murad MH. Health assessment of commercial drivers: a meta-narrative systematic review. BMJ Open 2014;4:e003434.

A22. Campbell R, Pound P, Pope C, Britten N, Pill R, Morgan M, et al. Evaluating meta-ethnography: a synthesis of qualitative research on lay experiences of diabetes and diabetes care. Soc Sci Med 2003;56:671-684.

A23. Campbell R, Pound P, Morgan M, Daker-White G, Britten N, Pill R, et al. Evaluating meta-ethnography: systematic analysis and synthesis of qualitative research. Health Technol Assess 2011;15:1-164.

A24. Thorne S, Paterson B, Acorn S, Canam C, Joachim G, Jillings C. Chronic illness experience: insights from a metastudy. Qual Health Res 2002;12:437-452.

A25. Finfgeld DL. Courage as a process of pushing beyond the struggle. Qual Health Res 1999;9:803-814.

A26. Sandelowski M, Voils CI, Barroso J. Defining and Designing Mixed Research Synthesis Studies. Res Sch 2006;13:29.

A27. Flemming K. The knowledge base for evidence-based nursing: a role for mixed methods research? ANS Adv Nurs Sci 2007;30:41-51.

A28. Sandelowski M, Voils CI, Leeman J, Crandell JL. Mapping the Mixed Methods-Mixed Research Synthesis Terrain. J Mix Methods Res 2012;6:317-331.

A29. Seymour J. Combined qualitative and quantitative research designs. Curr Opin Support Palliat Care 2012;6:514-524.

Appendix 2. Some articles related to qualitative research about health care services

Domain	Related articles*
Preventive Medicine	Pharmacoepidemiology [B01-B02]<br>Occupation [B03]<br>Hospital Management [B04]
Clinical Medicine	Obstetrics [B05]<br>Hospice [B06]<br>Patient-Doctor relationship [B07]
Nurse	Diabetic care [B08, B09]<br>Insight into cancer [B10]
Health Promotion	Mental Health [B11]<br>School Health [B12-B15]<br>Health Program [B16-B20]<br>Vaccine Program [B21]
Health Policy	Policy Decision [B22]<br>Control Plan of Tuberculosis [B23]

* [Reference number]

B01. Pound P, Britten N, Morgan M, Yardley L, Pope C, Daker-White G, et al. Resisting medicines: a synthesis of qualitative studies of medicine taking. Soc Sci Med 2005;61:133-155.

B02. Teasdale E, Santer M, Geraghty AW, Little P, Yardley L. Public perceptions of non-pharmaceutical interventions for reducing transmission of respiratory infection: systematic review and synthesis of qualitative studies. BMC Public Health 2014;14:589.

B03. Ruggiero JS, Redeker NS. Effects of napping on sleepiness and sleep-related performance deficits in night-shift workers: a systematic review. Biol Res Nurs 2014;16:134-142.

B04. Mousazadeh Y, Jannati A, Jabbari Beiramy H, Asgharijafarabadi M, Ebadi A. Advantages and disadvantages of different methods of hospitals' downsizing: a narrative systematic review. Health Promot Perspect 2013;3:276-287.

B05. Boath E, Bradley E, Henshaw C. The prevention of postnatal depression: a narrative systematic review. J Psychosom Obstet Gynaecol 2005;26:185-192.

B06. Flemming K. The use of morphine to treat cancer-related pain: a synthesis of quantitative and qualitative research. J Pain Symptom Manage 2010;39:139-154.

B07. Santer M, Ring N, Yardley L, Geraghty AW, Wyke S. Treatment non-adherence in pediatric long-term medical conditions: systematic review and synthesis of qualitative studies of caregivers' views. BMC Pediatr 2014;14:63.

B08. Davidson MB. The effectiveness of nurse- and pharmacist-directed care in diabetes disease management: a narrative review. Curr Diabetes Rev 2007;3:280-286.

B09. Davidson MB. Detailed treatment algorithms for effective nurse- and pharmacist-directed diabetes care: a personal approach. Diabetes Educ 2009;35:61-71.

B10. Jayde V, White K, Blomfield P. Symptoms and diagnostic delay in ovarian cancer: a summary of the literature. Contemp Nurse 2010;34:55-65.

B11. Leamy M, Bird V, Le Boutillier C, Williams J, Slade M. Conceptual framework for personal recovery in mental health: systematic review and narrative synthesis. Br J Psychiatry 2011;199:445-452.

B12. Thomas J, Harden A, Oakley A, Oliver S, Sutcliffe K, Rees R, et al. Integrating qualitative research with trials in systematic reviews. BMJ 2004;328:1010-1012.

B13. Harden A, Brunton G, Fletcher A, Oakley A. Teenage pregnancy and social disadvantage: systematic review integrating controlled trials and qualitative studies. BMJ 2009;339:b4254.

B14. Bonell C, Harden A, Wells H, Jamal F, Fletcher A, Petticrew M, et al. Protocol for a systematic review of the effects of schools and school-environment interventions on health: evidence mapping and syntheses. BMC Public Health 2011;11:453.

B15. Jamal F, Fletcher A, Harden A, Wells H, Thomas J, Bonell C. The school environment and student health: a systematic review and meta-ethnography of qualitative research. BMC Public Health 2013;13:798.

B16. Liu J, Davidson E, Bhopal R, White M, Johnson M, Netto G, et al. Adapting health promotion interventions to meet the needs of ethnic minority groups: mixed-methods evidence synthesis. Health Technol Assess 2012;16:1-469.

B17. Neville LM, O'Hara B, Milat A. Computer-tailored physical activity behavior change interventions targeting adults: a systematic review. Int J Behav Nutr Phys Act 2009;6:30.

B18. Neville LM, Milat AJ, O'Hara B. Computer-tailored weight reduction interventions targeting adults: a narrative systematic review. Health Promot J Austr 2009;20:48-57.

B19. Neville LM, O'Hara B, Milat AJ. Computer-tailored dietary behaviour change interventions: a systematic review. Health Educ Res 2009;24:699-720.

B20. Thomas R, Perera R. School-based programmes for preventing smoking. Cochrane Database Syst Rev 2006;(3):CD001293.

B21. Mills E, Jadad AR, Ross C, Wilson K. Systematic review of qualitative studies exploring parental beliefs and attitudes toward childhood vaccination identifies common barriers to vaccination. J Clin Epidemiol 2005;58:1081-1088.

B22. Contandriopoulos D, Lemire M, Denis JL, Tremblay E. Knowledge exchange processes in organizations and policy arenas: a narrative systematic review of the literature. Milbank Q 2010;88:444-483.

B23. Noyes J, Popay J. Directly observed therapy and tuberculosis: how can a systematic review of qualitative research contribute to improving services? A qualitative meta-synthesis. J Adv Nurs 2007;57:227-243.
